# Supplementary material for: Allosteric role of the citrate synthase homology domain of ATP citrate lyase
Source: Nat Commun. 2023 Apr 19;14:2247. doi: 10.1038/s41467-023-37986-9 (PMC10115795; doi:10.1038/s41467-023-37986-9)
Supplement: Supplementary file 1 — Supplementary Information [file 41467_2023_37986_MOESM1_ESM.pdf]

Supplementary Information

Allosteric role of the citrate synthase homology domain of ATP citrate lyase

Xuepeng Wei [1,2], Kollin Schultz [3], Hannah L. Pepper [4,5], Emily Megill [4,5], Austin Vogt [1,2], Nathaniel W. Snyder [4,5] and Ronen Marmorstein [1,2,3]

[1] Department of Biochemistry & Biophysics, Perelman School of Medicine, University of Pennsylvania, Philadelphia, PA, 19104

[2] Abramson Family Cancer Research Institute, Perelman School of Medicine, University of Pennsylvania, Philadelphia, PA, 19104

[3] Graduate Group in Biochemistry & Molecular Biophysics, Perelman School of Medicine, University of Pennsylvania, Philadelphia, PA, 19104

[4] Department of Cardiovascular Sciences, Lewis Katz School of Medicine, Temple University, Philadelphia, PA, 19140

[5] Center for Metabolic Disease Research, Lewis Katz School of Medicine, Temple University, Philadelphia, PA, 19140

Correspondence to: Ronen Marmorstein at marmor@upenn.edu

This file includes:

SupplementarySupplementary Figures 1 to 8

a

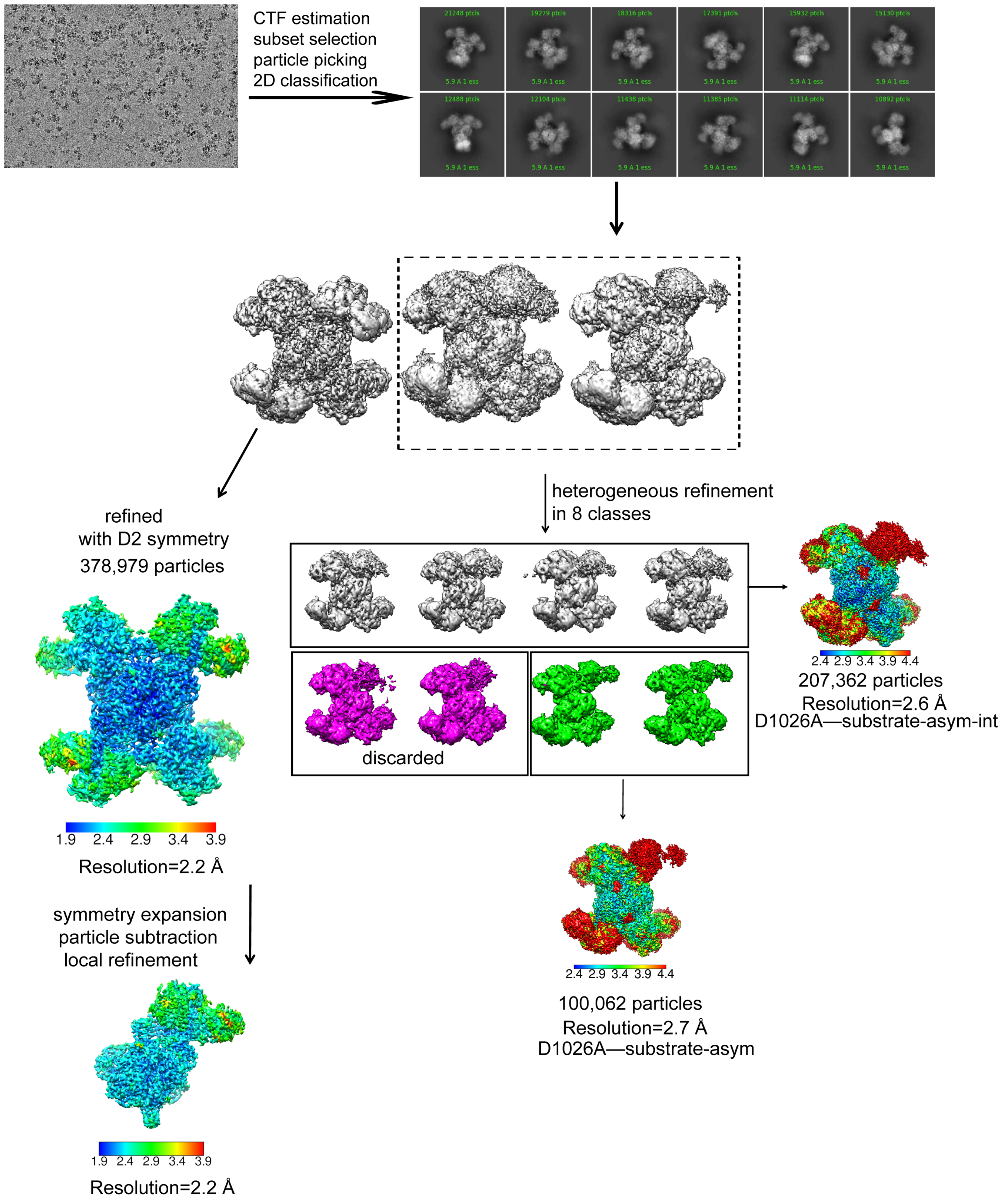

CTF estimation
subset selection
particle picking
2D classification
21248 ptcls
19279 ptcls
18316 ptcls
17391 ptcls
15932 ptcls
15130 ptcls
12488 ptcls
12104 ptcls
11438 ptcls
11385 ptcls
11114 ptcls
10892 ptcls
5.9 A 1 ess
refined
with D2 symmetry
378,979 particles
1.9 2.4 2.9 3.4 3.9
Resolution=2.2 Å
symmetry expansion
particle subtraction
local refinement
1.9 2.4 2.9 3.4 3.9
Resolution=2.2 Å
heterogeneous refinement
in 8 classes
discarded
2.4 2.9 3.4 3.9 4.4
207,362 particles
Resolution=2.6 Å
D1026A—substrate-asym-int
2.4 2.9 3.4 3.9 4.4
100,062 particles
Resolution=2.7 Å
D1026A—substrate-asym

b

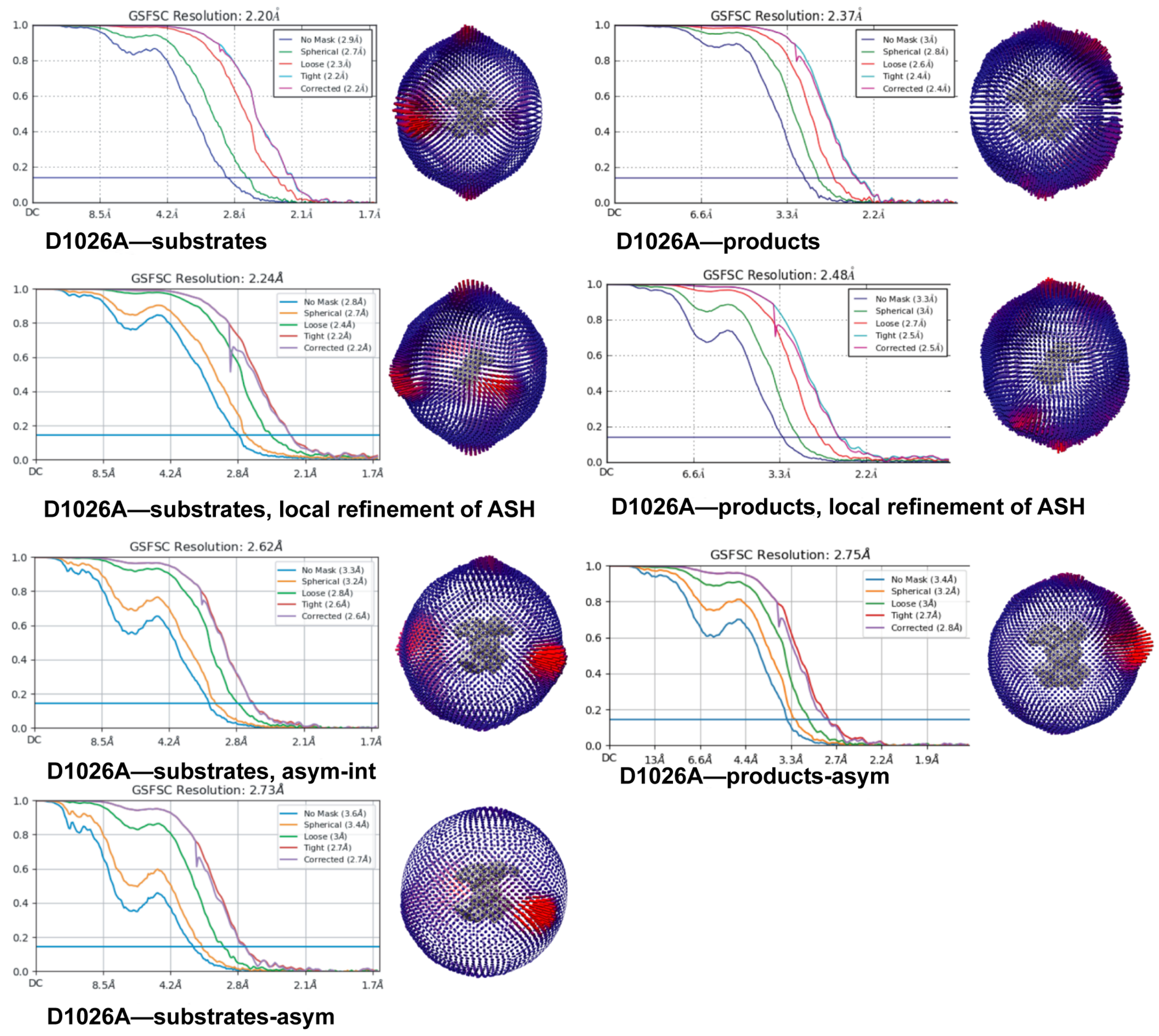

Supplementary Figure 1. **Workflow, Fourier Shell Correlation (FSC) curves and particle image orientation distribution for structures and maps.** (a) Workflow of image processing of ACLY-D1026A—substrates structure. Workflow for other structures were similar. (b) Fourier shell correlation (FSC) curves and particle image orientation distribution (build file) for refined maps.

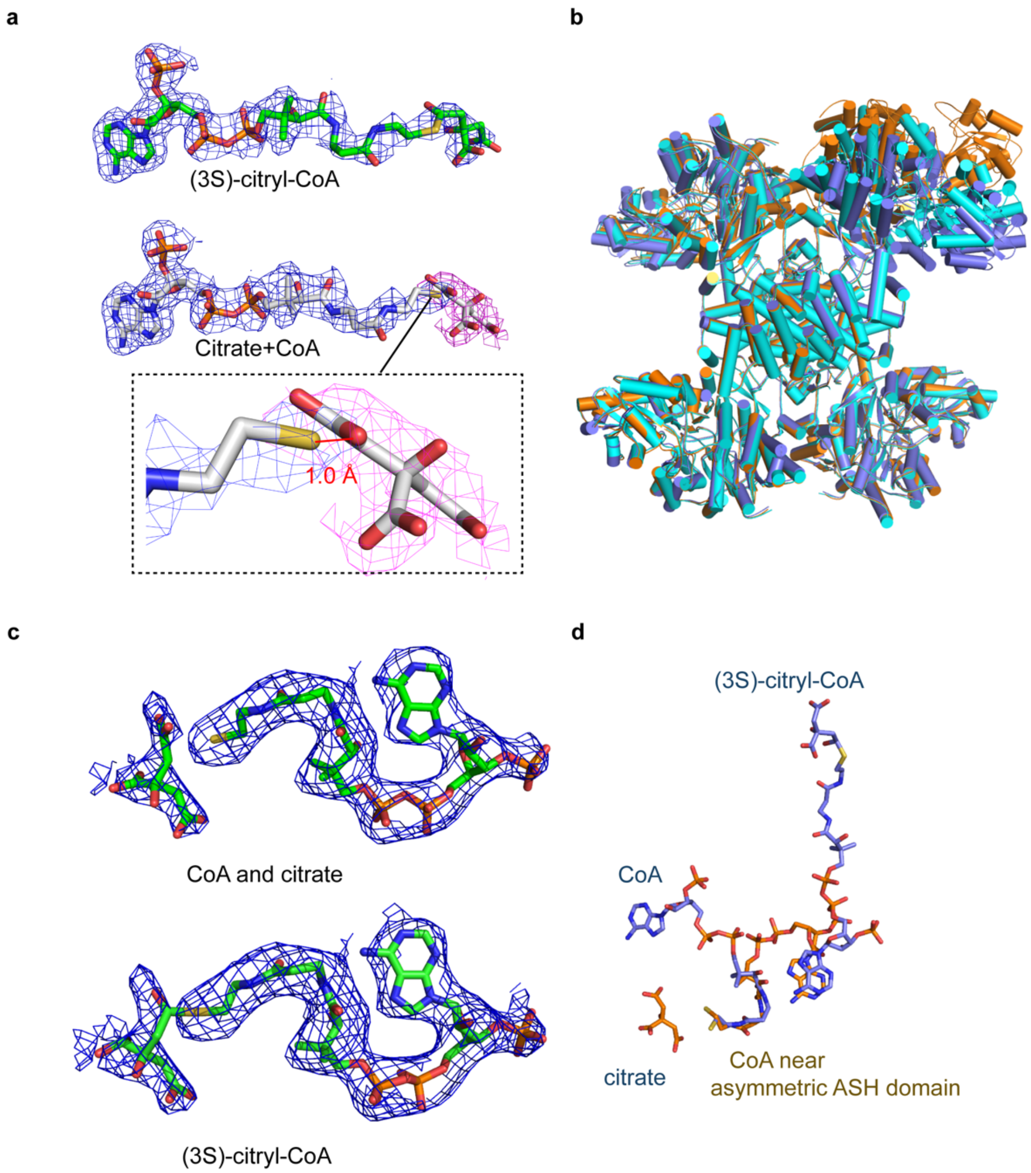

Supplementary Figure 2. Comparison of structures of ACLY-D1026A with substrates in symmetric and asymmetric conformations. (a) Cryo-EM density of the bound ligand in ACLY-D1026A—substrates, with the ligand in the ASH domain assigned as (3S)-Citryl-CoA instead of CoA and citrate, which would form a significant atomic clash as illustrated in the insert. (b) Cartoon of structural overlay highlighting a rotation of the asymmetric ASH subunit by ~ 40 degrees when comparing structures of ACLY-D1026A—substrates with ACLY-D1026A—substrates-asym. (c) Cryo-EM density proximal to the asymmetric ASH domain can be modeled bound to the CSH domain in a non-productive conformation as either CoA + citrate (top) or (3S)-citryl-CoA (bottom). (d) Overlay of ASH domain-bound (3S)-citryl-CoA and CSH domain-bound non-canonical CoA from the symmetric ACLY-D1026A—substrates structure with the CSH domain-bound non-productive CoA near the ASH domain of asymmetric ACLY-D1026A—structure.

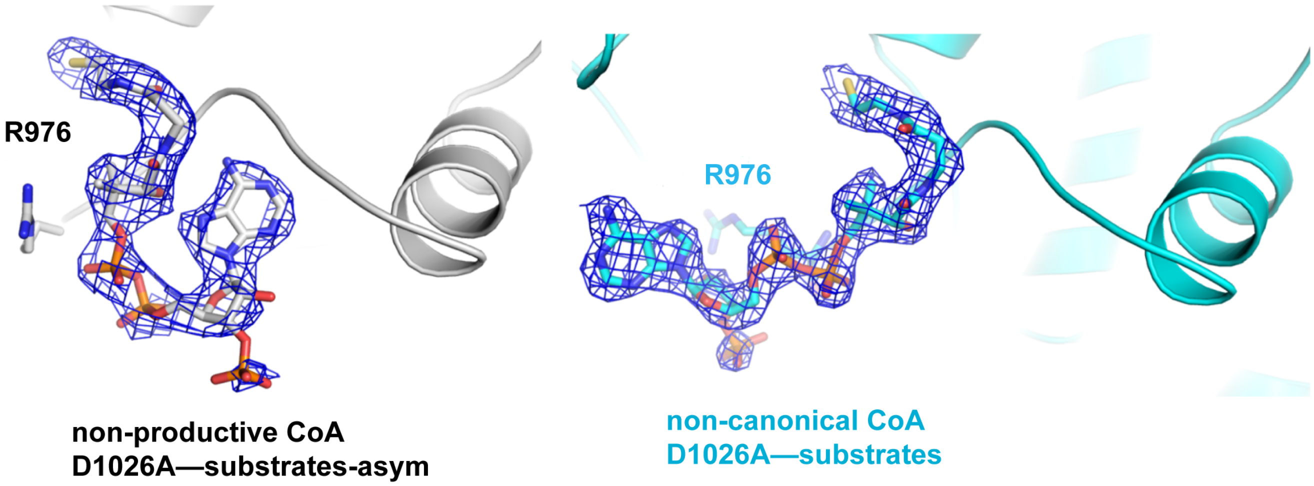

Supplementary Figure 3. **Comparison of the non-productive CoA and non-canonical CoA binding sites in ACLY-D1026A—substrate structures.** Non-productive CoA is from ACLY-D1026A—substrates-asym (similar to non-productive CoA in ACLY-WT with CoA (PDB 6UUZ)) and non-canonical CoA is from ACLY-D1026A—substrates. The reorientation of R976 to accommodate the bound non-canonical CoA molecule in the ACLY-D1026A—substrate (aqua) as opposed to the from ACLY-D1026A—substrates-asym (gray) is highlighted. The cryo-EM maps are contoured at 3 sigma.

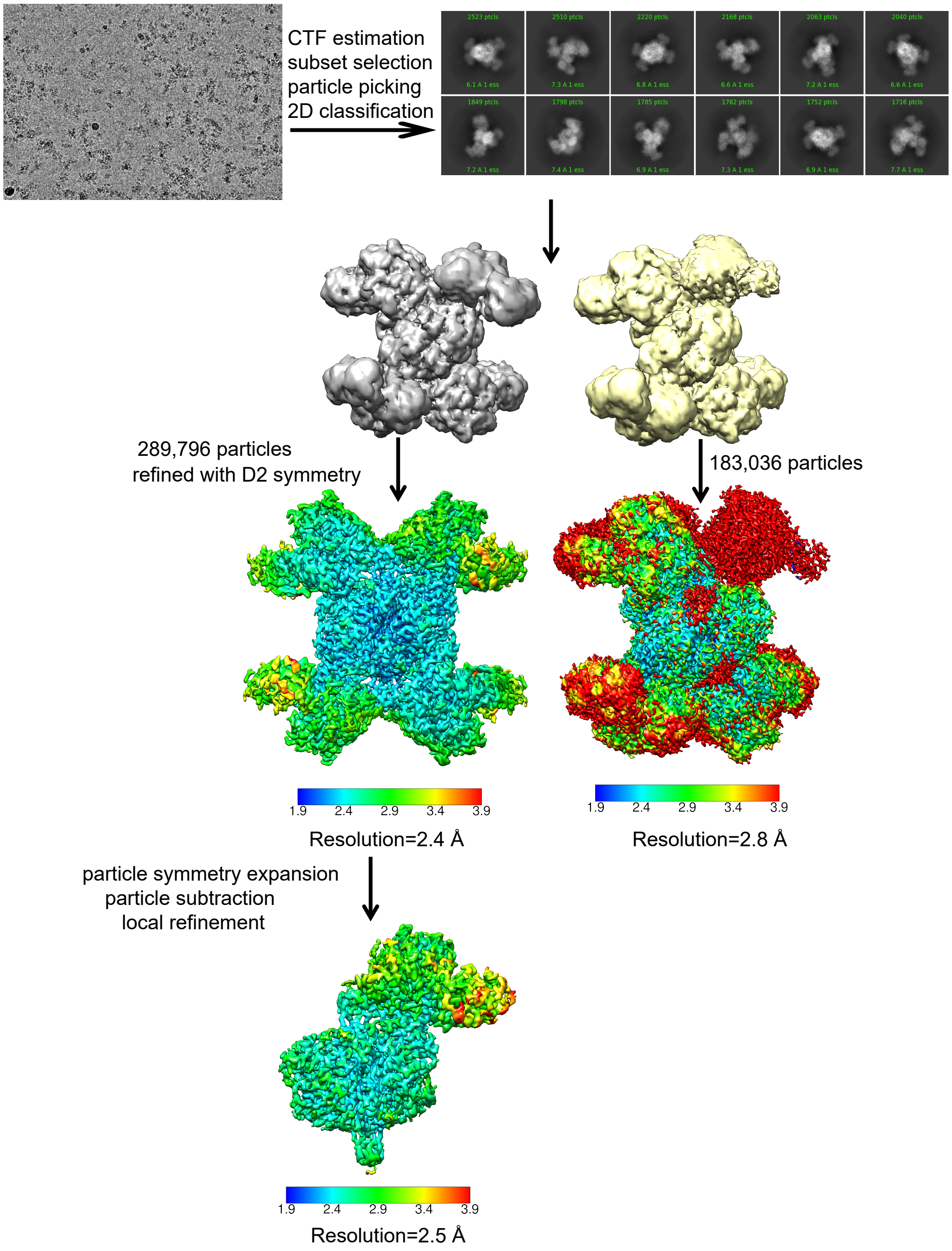

Supplementary Figure 4. Workflow for image processing of ACLY-D1026A—products structure.

Workflow was similar to ACLY-D1026A—substrates structure as detailed in Supplementary Figure 1.

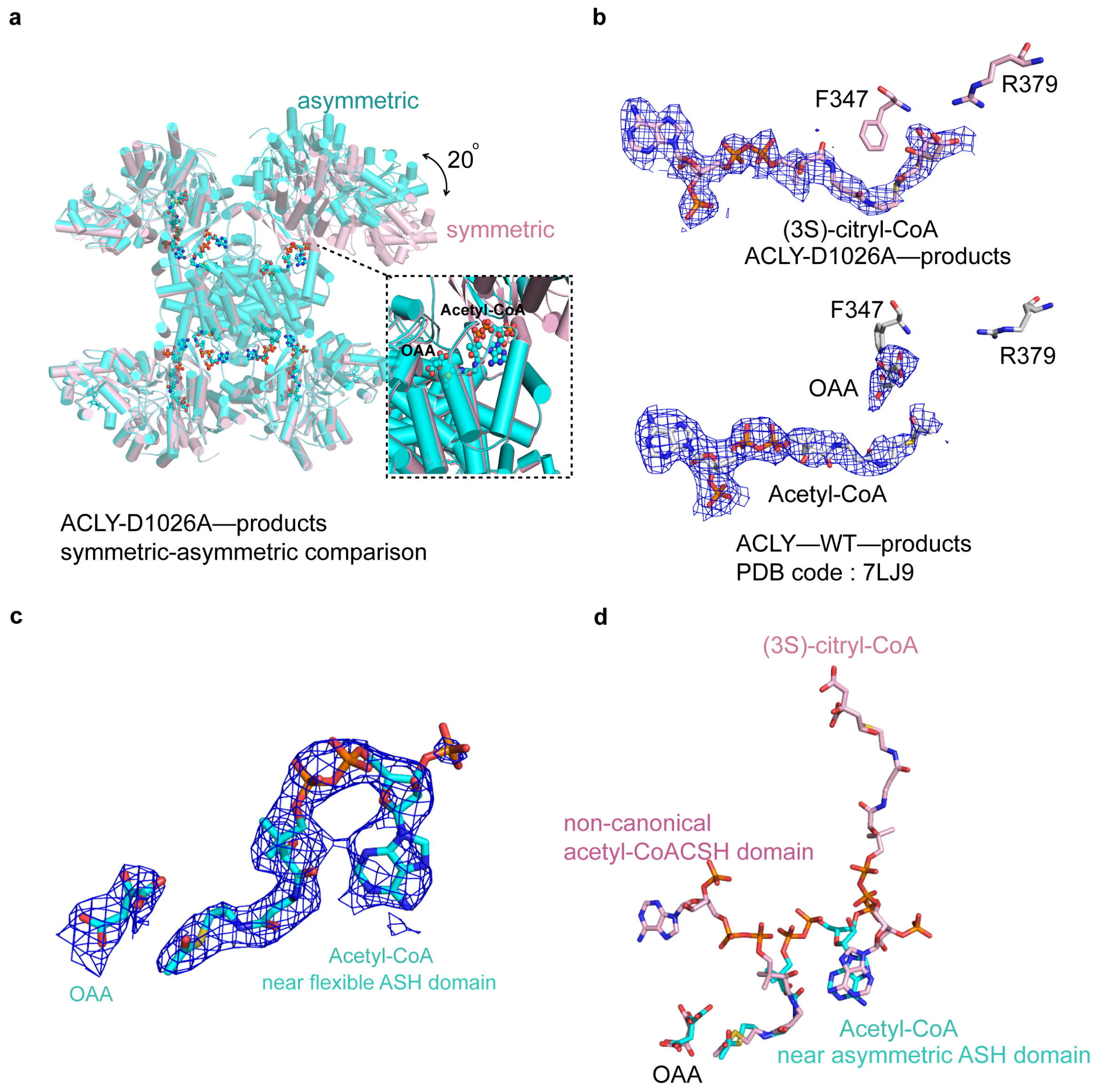

Supplementary Figure 5. **Comparison of structures of ACLY-D1026A with products in symmetric and asymmetric conformations.** (a) Cartoon of structural overlay highlighting a rotation of the asymmetric ASH subunit by ~ 20 degrees. The insert highlights the bound unloaded acetyl-CoA (and OAA) proximal to the asymmetric ASH subunit. (b) Cryo-EM density of (3S)-citryl-CoA from ACLY-D1026A—products (top) and acetyl-CoA and OAA from ACLY-WT—products structures (bottom), highlighting residues that undergo conformational changes that accommodate the transition from(3S)-citryl-CoA to acetyl-CoA + OAA. (c) Cryo-EM density of acetyl-CoA + OAA proximal to the asymmetric ASH domain and CSH domain-bound of the asymmetric ACLY-D1026A—products structure. (d) Overlay of ASH domain-bound (3S)-citryl-CoA and CSH domain-bound non-canonical acetyl-CoA from the symmetric ACLY-D1026A—products structure with the CSH domain-bound unloaded acetyl-CoA near the ASH domain of asymmetric ACLY-D1026A—products structure.

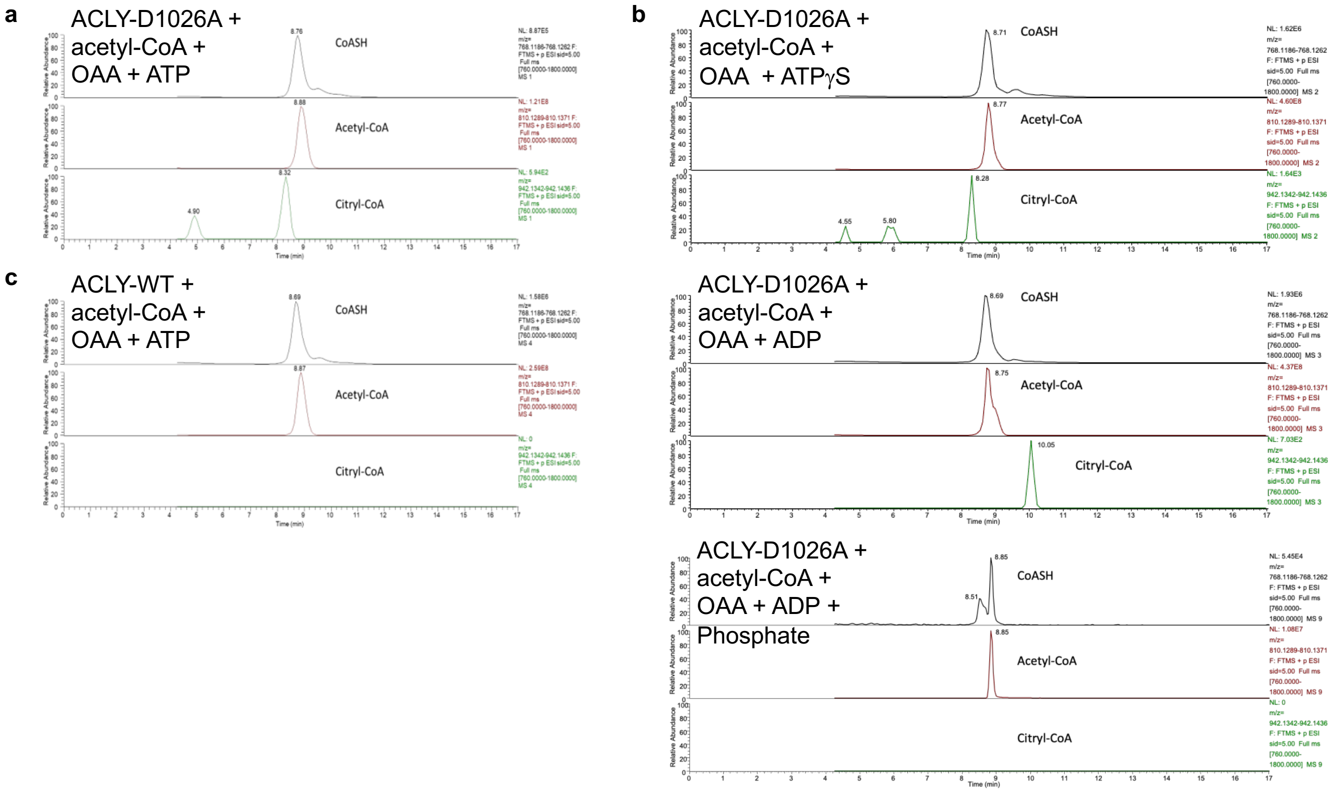

Supplementary Figure 6. **Identification of acyl-CoAs by ACLY-D1026A and ACLY-WT.** (a) Production of citryl-CoA (retention time 8.3, $[M+H]^+$ ion at m/z 942.1389) by ACLY-D1026A. (b) Production of citryl-CoA by ACLY-D1026A incubated with acetyl-CoA, OAA and ATPγS, but not with replacement of ATP/ ATPγS with ADP or ADP + phosphate. (c) ACLY-WT incubated with acetyl-CoA, OAA and ATP does not form citryl-CoA. Details are described in legend to Figure 5.

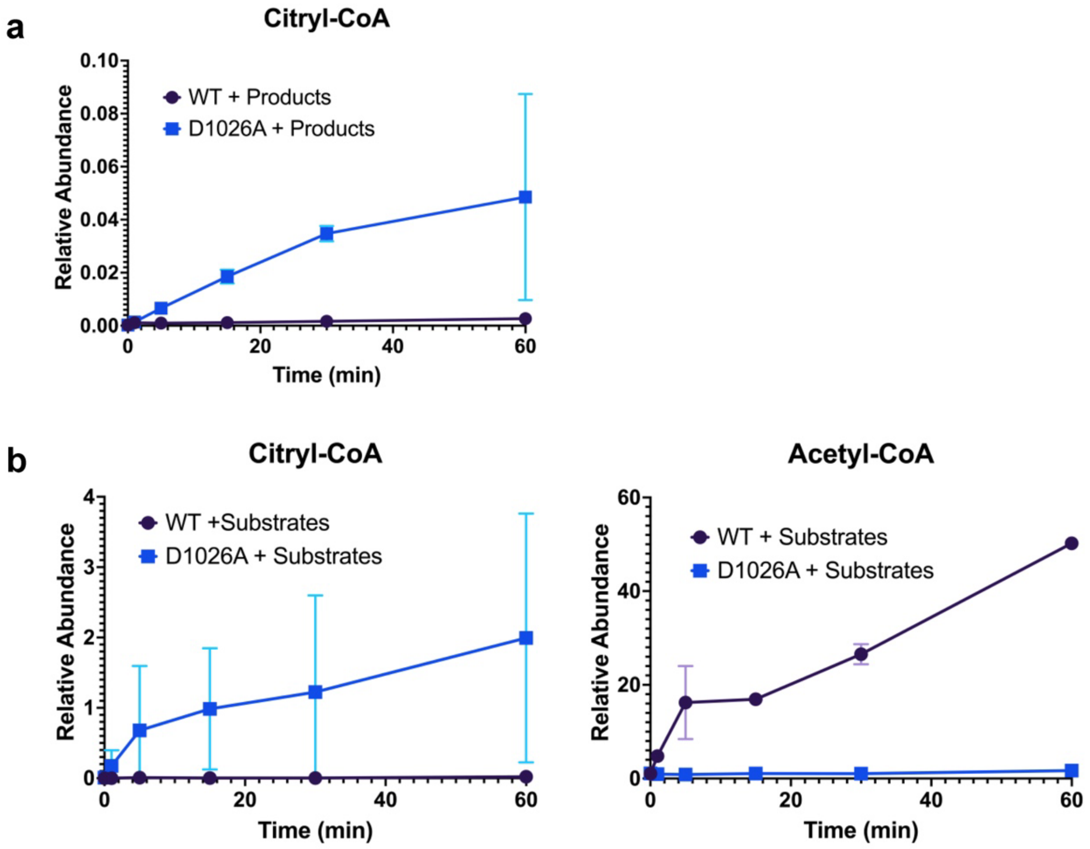

Supplementary Figure 7. **Time course of citryl-CoA generation by the ACLY-D1026A in the presence of products and substrates.** (a) Production of citryl-CoA by ACLY-D1026A, but not ACLY-WT, incubated with acetyl-CoA and OAA products and ATP. Production of CoA could not be evaluated due to CoA contamination of acetyl-CoA stock solutions obtained from vendors. (b) Production of citryl-CoA by ACLY-D1026A, but not ACLY-WT, incubated with CoA, citrate and ATP substrates. Production of acetyl-CoA by ACLY-WT, but not ACLY-D1026A, is also shown. All reactions were carried out in triplicate and averages with standard deviations are shown. $^{13}C_3$ $^{15}N_1$-HMG-CoA was used as a surrogate internal standard to derive relative abundance of citryl-CoA.

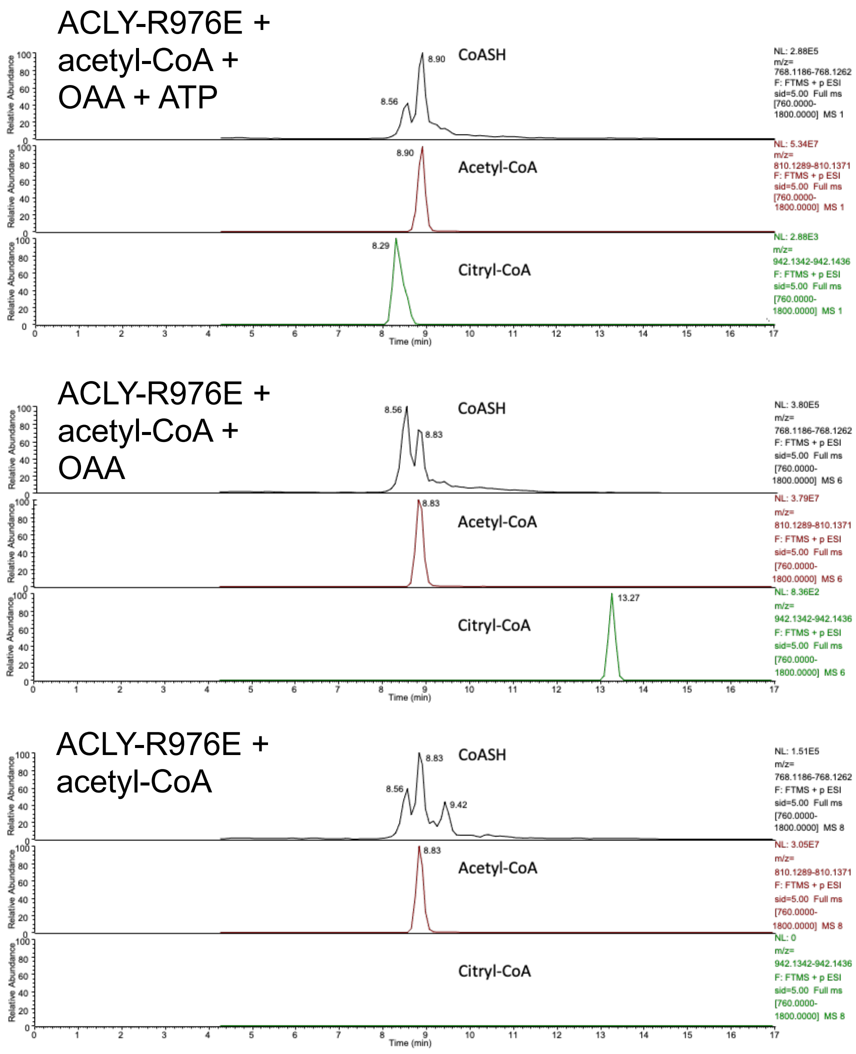

Supplementary Figure 8. **Identification of acyl-CoAs by ACLY-R9766E.** Production of citryl-CoA (retention time 8.3, [M+H]+ ion at m/z 942.1389) by ACLY-R976E in the presence of acetyl-CoA, OAA and ATP (top), but not in the absence of ATP (middle) or in the absence ATP and OAA.
